# Cross-cultural code-switching – the impact on international medical graduates in New Zealand

**DOI:** 10.1186/s12909-023-04900-2

**Published:** 2023-12-05

**Authors:** Mariska M. Mannes, Davinia J. Thornley, Tim J. Wilkinson

**Affiliations:** 1 School of Social Sciences, University of Otago Dunedin, PO Box 56, Dunedin, 9054 New Zealand; 2 School of Social Sciences - Media, Film and Communication, University of Otago Dunedin, Dunedin, New Zealand; 3 Department of Medicine, University of Otago Christchurch, Christchurch, New Zealand

**Keywords:** Values, Cross-cultural code-switching, Vulnerability, International medical graduates, New Zealand

## Abstract

**Background:**

New Zealand relies on International Medical Graduates (IMGs); however, the retention of IMGs is not optimal. This research uses a lens of cross-cultural code-switching to explore how professional and cultural differences impact on International Medical Graduates’ (IMGs’) journeys to practise effectively and remain in New Zealand.

**Methods:**

Utilising theory-informing inductive analysis within a constructivist approach, framework analysis was conducted following 14 face-to-face interviews with IMGs. The analysis then explored the degree to which their experiences could be explained by cross-cultural code-switching’s psychological challenges (authenticity, competence, and resentment).

**Results:**

Analysis showed there was an expectation for IMGs to code-switch. The greater the cultural and professional difference of IMGs (compared to New Zealand), the greater the intensity of psychological challenges experienced when switching. Moreover, IMGs received minimal support, making it difficult to overcome psychological challenges, especially the competence challenge. This led to feelings of frustration and vulnerability. Code-switching could also explain why complaints about IMGs were more likely when IMGs were stressed or tired.

**Conclusion:**

Cross-cultural code-switching can be used to explain and identify how cultural differences cause psychological challenges. These findings inform how programmes can better support IMGs in orientation and ongoing training. Additionally, establishing, and allocating IMG cultural mentors would assist in addressing IMGs’ vulnerability and isolation. With this support, the journey may prove more manageable and encourage IMGs to continue practising in their adopted country.

## Introduction

Like many OECD countries, New Zealand relies on International Medical Graduates (IMGs) [1] to fill medical practitioner shortages so retention is vital. IMGs are doctors who obtained their primary medical qualification in a country other than New Zealand [2]. New Zealand accepts IMGs from comparable and incomparable health systems. Comparable systems are measured by the likeness of a country’s health environment and the similarity of their medical registration system compared to New Zealand [3]. The Medical Council of New Zealand (MCNZ) currently lists 24 countries as having a comparable system; all others are considered incomparable and are likely to be culturally different to New Zealand.

To practise effectively, IMGs need to adapt both professionally and culturally when they migrate. The greater the differences between the culture of New Zealand and those of IMGs, the greater the amount of adaptation needed. Engaging with these differences and switching to a new way of doing things adds new experiences [4]; however, where the switch conflicts with the IMG’s internal beliefs, they are likely to experience some emotional or psychological stress. Prior to data collection and analysis, we considered a range of theories to explore these issues, such as acculturation and cultural value dimensions theory [5–10]. As the analysis proceeded, we drew on the methodology of Varpio et al. where theory was used as an interpretive tool [11]. The decision on which theory informed the final interpretation of the data was only finalised during the data collection and analysis cycles. It was at this stage that we identified cross-cultural code-switching [12] as a useful theoretical framework to understand how people navigate such new cultural environments.

Cross-cultural code-switching ‘is the conscious act of deviating from one’s native, default cultural code of interaction in order to meet or match expectations for behavior in a foreign cultural setting’ (13 p. 624). Code-switching originated in linguistics and is the process of switching from one linguistic code to another depending on the social context or conversational setting [13]. Molinsky expanded the code-switching concept, related it to cultural norms and coined the term cross-cultural code-switching [14]. He identified three psychological challenges associated with cross-cultural code-switching: authenticity, competence, and resentment. These challenges register the impact of cultural differences and the emotions triggered when expected to adapt. Additional cross-cultural theories including cultural value dimensions [5, 7, 15] and acculturation [8, 9, 16] underpinned the research in understanding the cultural journey of IMGs practising in New Zealand.

Within this paper, cultural code is the implicit and/or explicitly learned values, beliefs, behaviours, and practices that form the norms from which individuals operate and determine their worldview [5, 10, 17]. Similarly, professional values are learned, guiding principles that influence an individual’s behaviour at work. These influences might include the tacit norms of the workplace, management policies and legal obligations.

New Zealand’s health system is based on the patient-centred model [18, 19]. Codes, such as the New Zealand Health and Disability Act [20], the New Zealand Health Information Privacy Code 2020 [21] and New Zealand’s founding document the Treaty of Waitangi - Te Tiriti o Waitangi [22], also influence and guide practice. Thus, even IMGs from comparable health systems need to adapt the way they practise, more so for those from incomparable systems, and those who are culturally diverse.

Identifying cultural differences is essential to recognising potential challenges IMGs may face on their journey to practising effectively. Therefore, this research project included two phases: the first phase was quantitative and identified the cultural and professional values of doctors (both New Zealand and IMG) practising in New Zealand and explored the challenges New Zealand doctors faced working alongside IMG colleagues [23], the second phase, as presented in this article, was qualitative and explored the challenges IMGs face via interviews. The data were analysed using theory-informed inductive analysis.

Common challenges IMGs face, as identified in previous research, include communication, adapting to a patient-centred model, cultural differences, and the re-registration process [24–28]. Less is known about how these challenges impact the psychological well-being of IMGs.

Hence, this research aims to explore the degree to which the psychological challenges of cross-cultural code-switching due to professional and cultural differences might impact IMGs’ journeys to practising effectively and remaining in New Zealand.

## Methods

The research drew on semi-structured face-to-face interviews with 14 IMGs to understand their challenges while practising in New Zealand.

In this study we used a constructivist research approach utilising the process of theory-informed inductive analysis [11, 29, 30] via framework analysis [31]. In the initial phase we drew on a range of cross-cultural theories [5, 7, 10, 32, 33] to inform the research, and these theories were kept in mind during data analysis. Thematic analysis was used to find patterns, and, as outlined in the introduction, the decision on which theory informed the final interpretation of the data was only finalised during the data collection and analysis cycles [11]. This led us to identify cross-cultural theory as the most appropriate to explain the participants’ experiences.

### Study participants and recruitment

The focus of this study was on IMG doctors who had been qualified for over two years before arriving in New Zealand and were in current practice in New Zealand for more than one year.

Ethics approval was obtained from the University of Otago Human Ethics Committee (Ref no: 19/071) and Ngāi Tahu Research Consultation Committee [34]. All research was undertaken in accordance with the methodological framework that was submitted and approved. The main ethical considerations were the anonymity and vulnerability of the participants. However, these were mitigated by pre-briefing participants, gaining informed consent, and having debriefing strategies and support in place.

IMG doctors were recruited from those who indicated their interest during phase 1 [23], invitations were also included in the Royal New Zealand College of General Practitioners online newsletter, and others were recruited via snowballing. Upon agreeing to participate, the IMG was sent a list of very broad interview questions asking them to reflect on the differences between the health system they came from compared to the New Zealand one, for example, practice differences, cultural differences, and challenges faced because of these differences. Sending the questions in advance allowed IMGs to reflect on their journey and the information they might share. This was important to lessen any vulnerability, especially if they were experiencing challenges that had resulted in negative consequences, were struggling to ‘fit in’, or were experiencing stress, discrimination, or isolation.

### Data collection

Interviews were held at a time and place convenient to the participant. The interviews were conducted in person by the first author, in English, audio recorded, and were, on average, one hour in duration. Time was not restricted to allow participants to share as much information as they wished at their own pace. Except for one, participants were not known to the interviewer. Participants gave written informed consent beforehand and were reminded that they could turn off the recorder at any time. Time for a debrief was allowed if the information they shared had caused the participant distress. Support sources were also provided in the information sheet.

At the end of each interview, the interviewer summarised what was discussed and checked with the IMG that this represented a true account of the discussion. Results of the preliminary thematic analyses were shared with participants, and they were invited to share any further comments or clarifications. No clarifications were made. Participants were appreciative of being kept informed and were interested in the results.

Using Guest et al’s [35] thematic saturation approach and a base size of six interviews, we reached the < 5% new information threshold at 8^+2^ interviews. Four further interviews were conducted to ensure diversity of IMGs was reached; no further themes were found. Data collection was conducted from November 2020 to August 2021.

### Data analysis

Interviews were transcribed verbatim; any personally identifiable information was removed. All authors had access to the interview transcripts. The interview data were then analysed iteratively by the first author, using thematic analysis [30], concurrently with data collection. Each transcript was reviewed and analysed, identifying responses that informed cross-cultural values theory and identifying themes through patterned responses. As the inductive analysis proceeded, and discussion among the researchers developed, we recognised that the findings fitted best with cross-cultural code-switching. Following this, the final phase involved clarifying the relationship between the themes and cross-culture code-switching theory. Therefore, cross-cultural code-switching theory was applied as an interpretive tool [11, 12].

Within this theory, Molinsky proposes three psychological challenges: authenticity, competence, and resentment challenge [12]. The authenticity challenge covers emotions experienced when the new expected behaviour conflicts with one's original internal beliefs and value system; the competence challenge involves the struggle people face when they lack the knowledge and skills to function effectively in the new environment or adapt; and the third challenge, resentment, generates the feeling that adapting one’s cultural behaviour is an imposition, causing anger and frustration. These three challenges were used to interpret the data/themes.

Finally, all authors worked together to discuss the themes, interpretation and analysis and agree on findings. These meetings were essential to reflect on interpretations, and to discuss individual perspectives. This was especially useful in view of the diverse perspectives brought by the research team: one team member teaches cultural competency modules to health practitioners; another has extensive experience in conducting cross-cultural research; and the other is a New Zealand medically qualified doctor with interests and expertise in education. Additionally, as many experiences shared by the IMGs were emotional, discussions were vital to separate emotional perspectives from the challenges IMGs faced due to cross-cultural code-switching and adapting to the New Zealand health system.

## Results

The IMGs comprised six specialists, five general practitioners, and three registrars. Seven IMGs identified as female and seven as male. They came from the United Kingdom, the United States of America, South Africa, South America, and South and Southeast Asia. This sample included IMGs from both comparable and incomparable systems. The time participating IMGs had been practising in New Zealand ranged from one year to over ten years. Table 1 provides context information regarding gender, the year the IMG qualified as a medical doctor, and their time practising in New Zealand. To protect the anonymity of participants no further demographic information can be given.

**Table 1:** IMG demographic information

Year received medical qualification	Years practising in NZ	Gender
2011	2–5	Female
1992	10+	Female
1994	2–5	Female
1996	10+	Female
2014	2–5	Female
1994	2–5	Female
1999	2–5	Female
2001	< 2	Male
2001	< 2	Male
1989	10+	Male
2016	< 2	Male
1996	10+	Male
1994	< 2	Male
1990	10+	Male

The results from the interviews are grouped into four main themes: (1) expectations to code-switch, (2) communication, (3) adapting to practise, and (4) the consequences of cross-cultural code-switching. Within each of these themes the psychological challenges of authenticity, competence, and resentment are explored.

### Expectations to code-switch

Analysis of all interviews identified that the pressure IMGs experienced could be explained by code-switching to the way things were done in New Zealand. Additionally, IMGs recognised culture impacted practice and interactions with colleagues and patients. As these two IMGs explained,

‘I have this strong perception of how much [health] is based on cultural stuff … depends on what our clients expect, where clients can be supported, who is supposed to support them … what we can offer … so this forces me to be a doctor who’s completely different than I was in my country.’ [IMG 2].

‘You are expected to know the culture, you are expected to behave as if you were kiwi [New Zealander]. So, when you don’t people don’t like it.’ [IMG 3].

While code-switching was expected, it was made more difficult due to the lack of orientation, contributing to IMGs experiencing the competence challenge. Orientation to the health system and obligations under te Tiriti o Waitangi was given, but very little was offered regarding the wider New Zealand culture and context. Without this it was difficult for IMGs to find the skills within them to adjust, yet they were expected to anyway.

‘And when the person has no idea how they should behave, if they behave in a way which is good for them, it’s not good for them in a way that’s good for them here, and they’ll be confused, and they don’t understand.’ [IMG 5].

### Communication

Differences in communication styles challenged IMGs. Those with a more direct style struggled with the less direct style of their New Zealand colleagues. It left them uneasy, not knowing where they stood, and led them to being misunderstood by colleagues resulting in frustration and even becoming the subject of complaints. IMGs had to learn and adjust their way of communicating. As IMGs described,

‘I’m still trying to learn that it’s [directness] not always the best way. It’s just so frustrating because it just comes out … It’s kinda hard, because it’s part of your person, it’s part of your culture … it’s gotten me in trouble.’ [IMG 14].

‘When it happened, it was taken as a personal attack by my practice manager. I thought it was constructive feedback on some of the things that were happening to me … the way you say things really matter … the way I do things might not be to their liking, but they don’t say anything because they think they will hurt my feelings, but then it comes out in the most awkward way … I had to mend fences with my boss.’ [IMG 1].

As well as experiencing the competency challenge due to differences in communication styles, some IMGs also experienced the authenticity challenge. These IMGs struggled not only with having to adjust their communication style— which led to frustration— but also felt it went against what they believed, by not communicating directly and honestly, for example:

‘But the lack of directness, this false toxic positivity, when something is really wrong … we know it’s not working … but then everyone afterwards will go away complaining quietly. So, in terms of me wanting to be the best doctor I can be, this system is not conducive to that – because of the system and those cultural aspects.’ [IMG 11].

‘Here you have to sugar coat. I do not agree with it. Where I come from the dryer you are, the better you are, because you are not hiding anything. Here you must sugar coat, it’s like a new language, so I struggle, I try to copy but then I don’t know if it is the right occasion … sometimes people feel confronted if you ask for clarification … they [workplace] sent me on a communication course.’ [IMG 3].

IMG 3 went on to explain their strategy of overcoming the competency challenge,

‘I’m more mindful now of checking in or understanding, taking more time, using more silence, taking time to understand what the other person is requesting or asking or expecting’.

It was not only the communication style of the wider New Zealand population that caused the competency challenge for IMGs but also that of Māori (indigenous people of New Zealand) patients. All IMGs attended training introducing them to the Māori culture; however, this did not prepare IMGs for interacting with Māori patients and whānau (extended family). IMGs found many Māori patients interacted and communicated differently to the wider New Zealand culture, creating some confusion or anxiety for most IMGs. Thus, IMGs found different competencies were needed to interact effectively and respectfully with Māori.

‘What I found difficult was that whole thing about the way you structure your question, because it would often get a response, certainly from Māori and Pacifica, that they think you want to hear not necessarily what they are thinking, or what they want to say. You ask them if they’re okay and they say, “Yeah yeah”, when they’re actually not. So, trying to tease out the truth without accusing them of lying. Over the years I have developed a way that assists communication from my own perspective.’ [IMG 7].

‘I obviously try and respect as much as I can, everybody’s culture here, but there were some things that were personally conflictual for me, which was, for example, the karakia’ (incantations used to invoke spiritual guidance and protection) [IMG 11].

Furthermore, as part of their code-switching journey, many IMGs learnt and attempted some phrases in te reo Māori (the Māori language) to support a better cultural connection. While most commented they felt awkward with the pronunciation, they found when they used some te reo Māori there was a better connection. As one IMG pointed out, they were unsure if the switch would be accepted as genuine by the Māori person, for example,

‘I always wonder if I say “kia ora”, “ata mārie” (Māori greeting) to someone they’re going to turn and say, “who are you to talk to me like that, you’re not a New Zealander, not Māori, are you trying to pretend”. But in fact, it’s the opposite they’re pleased when you make an effort in te reo with them. So, my confidence is growing even though I’m probably mangling the pronunciation.’ [IMG 10].

### Adapting to practise

Different ways of interacting due to the New Zealand health obligations caused IMGs to experience the competency challenge, resulting in feeling pressure and anxiety. Expectations of practitioner roles and interactions with patients was different; IMGs needed time to understand their role and expectations, but this time was not available.

‘Here there are people who have roles that I don’t know what they are for, and I have to interact with them. And there are some professions that we don’t have in my country and others that we have that we don’t have here. Probably the same function is allocated to another nomination, in some part of the role but not exactly the same.’ [IMG 2].

Moreover, five IMGs discussed that within the system they came from, it was normal to include family in healthcare for consultations, decision-making, and asking for support regarding the patient; these IMGs had some resonance with Māori patients and whānau. The other IMGs, they had to learn a new way of interacting, as one IMG explained,

‘And so, yes, I am still, on a journey to understand more clearly. Because my view of medicine I was brought up with is to simply treat the illness … However, my own inclination is to treat the person. But the Māori condition seems much more to treat the whānau, the community. You know that the person might be who’s in front of you, but actually that person is, is the kind of tip of a very large iceberg of other people who are also involved. And if you don’t involve those people, then you’re missing something … you know, that model of practicing medicine is unusual for me.’ [IMG 10].

However, IMGs did find it difficult not to be able to call on the patient’s family for support. Thus, they needed to become comfortable consulting with the individual only. Upskilling to an almost opposite way of practising created some distress and anxiety for IMGs,

‘I usually relied on family support for clients. So, if I had a problem with clients, I was constantly calling families to come in and try to find strategies to take care of clients. It doesn’t work like that here. With some Māori families, it works like that, but with European, it doesn’t work like that at all. I have heard many times - I don’t want to know anything about my son or my daughter; it’s not my problem … If they [client] don’t want family involved, we cannot ask families to come to take care of them.’ [IMG 2].

‘In [country] if you suspect there is a serious diagnosis, you don’t tell the patient, you will tell the patient’s relatives about it. … And, if, unfortunately, something serious is being suspected, then the doctor will ask the patient actually to go out and wait in the waiting room and talk to the relative about the diagnosis; no one would break this rule.’ [IMG 4].

IMG 4 went on to explain that overcoming the competency challenge is easy when what you are expected to adjust to aligns with your values,

‘In my GP training … the art of breaking the bad news. I loved it, and it was fascinating, it is so new to me. I learned it quite well. You know, sometimes there’s something new, if it is not acceptable to you, then you don’t really learn it very well, but for some reason, it was so acceptable to me, I just felt that this is the right way of doing it.’

### The consequences of cross-cultural code-switching

IMGs felt they were being asked to become a different doctor with little regard for the knowledge they brought. IMGs admitted they expected differences and would need to adjust but did not expect a lack of support or interest from their New Zealand counterparts.

‘You should value your experience because although you may downplay it to these people, although they’ve done a fellowship somewhere for a year they’re still relatively, I could say, blinkered by their scope of practice … So don’t ever undersell yourself for what your breadth of your experience provides.’ [IMG 9].

IMGs are qualified and experienced, therefore, having to ask about ‘how things work around here’ or about a particular medical condition or treatment was difficult. There was a perception from IMGs that they would be perceived as incompetent, which created an unintentional barrier to cross-cultural code-switching and overcoming any competence challenges being experienced. Overcoming this required asking, especially as information was not forthcoming. Finding the confidence to ask left IMGs feeling vulnerable. The following alerts us to the amount of time it can take to build confidence to ask:

‘When I first arrived, I wasn’t empowered enough to actually ask them. Whereas now I will probably, I know my safe places … I’m older, more mature and I care a bit less about fitting in. I have social and financial security around me now. So, it doesn’t bother me, I’m happy to be vulnerable, but previously, I had this façade around me, and I didn’t want to come across as vulnerable cause I wasn’t sure how I’d be treated so I was initially just too worried. I will always be highly competent, highly capable, I would always portray that I was in control at all times. I just didn’t have the confidence to expose my lack of control or ask for help.’ [IMG 5].

‘I figured that if I keep on asking all the time, then I may not be eligible to stay. I kind of got lost at times, to someone else, it may have been enough to drive them away.’ [IMG 13].

Adding to the stress of finding information was then interpreting the information. All IMGs speak English, a requirement to practise in New Zealand; however, the mental fatigue of using English as a Second Language because of constant switches of language, was significant and added to the fatigue caused by code switching. It did not affect only the IMG at work but also spilled over into their private lives. An IMG stated:

‘English is not my first language, it takes a toll. So, the one that takes the toll is my husband because I say, “I’m not speaking English anymore, I’m out of work”. I have to, because he’s English … But it’s more than that, it’s going back home being very tired, it’s that mental fatigue, where you kind of say, I cannot think anymore.’ [IMG 3].

Under stressful situations it was difficult for IMGs to keep up the code-switch and they shared how they automatically resorted back to their engrained cultural behaviour, leading to complaints. As one IMG explains,

‘It’s exhausting! And then also the problem is that it’s a learned behaviour, when I’m stressed or exhausted or tired or sleep deprived or all of the above, I resort back to my normal behaviour. And then that will lead to some difficult conversations. So that’s the problem. Are you needing me to change fundamentally who I am, which means that I can’t carry on with that façade and then I am not true to myself.’ [IMG 5].

Building relationships with New Zealand colleagues and people was more difficult than anticipated. Most IMGs commented on a sense of isolation and a lack of social support. Those that were practising in an area close to other IMGs usually ended up socialising together, lessening the need to code-switch but bolstering the idea that IMGs stick together.

‘I would certainly suggest that you give your colleagues time, because they will be good friends and colleagues, but you are breaking into quite a small club who’ve known each other and probably been around the same med schools and hospitals for their entire professional life.’ [IMG 9].

‘We do meet up with the other doctors from other nationalities, we call ourselves the orphans. Easter weekend, all of us, like 12 of us, went to … just to have fun, so in a way social support.’ (IMG 13).

## Discussion

This study has revealed that IMGs do encounter psychological stress, due to cultural differences and because of their efforts to adapt to practising in a way expected in New Zealand. Such stresses can be explained, at least in part, through the theory of cross-cultural code-switching, as IMGs adapted to the psychological challenges of authenticity, competence, and resentment. Interestingly, whether the IMG had been in New Zealand for one year or ten years, their challenges and stressors were similar. This finding is important, as assumptions regarding integration and that the individual has adapted successfully may be false if the burden of cross-cultural code-switching remains with the IMG.

A sense of losing one’s identity, or part of it, is real for most IMGs, and the angst it causes should not be underestimated. Consistent with other studies [24, 27, 36, 37], this study also found that IMGs are confronted with many differences that, for some, have implications on their deeply held beliefs. These deeply held beliefs make cross-cultural code-switching more stressful, resulting in experiencing the authenticity challenge. Interestingly, for some IMGs in this study, connecting with Māori culture elicited mixed feelings and challenged their beliefs. Yet others found common ground with the whānau-based Māori approach. Identifying and linking the smallest commonalities with IMG beliefs may assist IMGs in transitioning to the new expected behaviour. However, the environment needs to be conducive to understanding the challenges IMGs face via relevant support resources that can abate the sense of identity loss.

Orientation to the broader cultural context of New Zealand was severely lacking. Other studies have also identified such a gap [38, 39] within their respective countries. Without knowledge of ‘how things work around here,’ it is difficult for IMGs to gain skills in competent practice, leaving them vulnerable and experiencing the competence challenge. This challenge can also lead to feeling like an imposter, as the IMG’s experience and knowledge is not as relevant in the new environment and can lead to the IMG believing they are not as competent as they believed. IMGs expect to and are willing to adapt, but a lack of cultural orientation and support leads to frustration. There is a strong argument for a comprehensive cultural orientation and ongoing support to assist IMGs in overcoming local challenges [24, 37, 40, 41].

Knowledge about cultural differences is critical to successful integration and practice. However, knowledge alone is not enough. IMGs need to transcend this knowledge by putting it into action and, at the same time, manage the anxiety that occurs because of the behaviour switch. Additionally, IMGs need the motivation and emotional strength to carry out the behaviour switch [4]. This is additional to all other challenges being experienced. When acquiring the ability to switch, knowledge of what is expected is the first step; then identifying whether the switch is right for the context and timing all come with practice [42]. This study found, however, that even with knowledge and practice, default cultural codes took over when IMGs were stressed or tired and, when this occurred, the likelihood of complaints increased. Furthermore, the expectation to continuously keep up the code switch, especially when IMGs are overwhelmed, adds another layer of pressure. Moreover, the cognitive load is greater when there are language differences. Conversely, switching was easier when things were going well, and IMGs had time to think about the switch.

Understandably, many IMGs experienced some form of resentment, whether it was from not being valued for their experience and knowledge or for having to adjust significantly by putting up a façade and not being accepted for themselves. These sentiments impacted on IMGs wellbeing and, for some, their desire to remain in New Zealand. For IMGs, cross-cultural code-switching is part of their journey. For their New Zealand counterparts and medical educators there is a genuine need to understand the impact of these psychological challenges and manage them.

A limitation to this study is that the cross-cultural code-switching lens was only identified following inductive analysis; therefore, IMGs were not asked specific questions regarding this topic. More specific questions might elicit further in-depth information of the impacts of switching.

Opportunities for further study lie in identifying additional cultural values that, when expected to code-switch, result in psychological challenges. Additionally, environmental factors that may lead to stress or tiredness and thereby cause IMGs to switch back to their default behaviour would be worthy of further exploration. This will be beneficial to identifying support IMGs need when practising in a country culturally diverse to their own. Identifying IMGs for whom the code-switch is more significant would be beneficial to developing support systems for those IMGs and identifying implications for practice.

## Conclusion

This study builds on the growing literature around the challenges IMGs face from the New Zealand context and adds a psychological perspective. Identifying the cultural differences that cause psychological challenges due to cross-cultural code-switching is vital for IMGs’ wellbeing and for identifying information needed to practise effectively. Programmes should be created addressing these challenges and included in orientation and ongoing curricula. Additionally, establishing cultural mentors for IMGs would assist in addressing IMGs’ vulnerability and isolation and help them feel valued. With this support, the journey may prove more manageable, encouraging IMGs to remain and strengthening the New Zealand population’s access to quality healthcare.

## Data Availability

The datasets generated and analysed during the current study are not publicly available to protect the anonymity of participants. Deidentified data are available from the corresponding author on reasonable request.
